# *Cis*-regulation of *IRF5 *expression is unable to fully account for systemic lupus erythematosus association: analysis of multiple experiments with lymphoblastoid cell lines

**DOI:** 10.1186/ar3343

**Published:** 2011-05-31

**Authors:** Elisa Alonso-Perez, Marian Suarez-Gestal, Manuel Calaza, Tony Kwan, Jacek Majewski, Juan J Gomez-Reino, Antonio Gonzalez

**Affiliations:** 1 Laboratorio Investigacion 10 and Rheumatology Unit, Instituto de Investigacion Sanitaria-Hospital Clinico Universitario de Santiago, Travesia Choupana sn, Santiago de Compostela E-15706, Spain; 2 Department of Human Genetics, McGill University, 1205 Dr Penfield Avenue, Montreal H3A 1B1, Canada; 3 Department of Medicine, University of Santiago de Compostela, San Francisco sn, Santiago de Compostela, E-15782, Spain

**Keywords:** systemic lupus erythematosus, *IRF5*, lymphoblastoid cell lines, *cis*-regulation, disease susceptibility, linear regression models

## Abstract

**Introduction:**

Interferon regulatory factor 5 gene (*IRF5*) polymorphisms are strongly associated with several diseases, including systemic lupus erythematosus (SLE). The association includes risk and protective components. They could be due to combinations of functional polymorphisms and related to *cis*-regulation of *IRF5 *expression, but their mechanisms are still uncertain. We hypothesised that thorough testing of the relationships between *IRF5 *polymorphisms, expression data from multiple experiments and SLE-associated haplotypes might provide useful new information.

**Methods:**

Expression data from four published microarray hybridisation experiments with lymphoblastoid cell lines (57 to 181 cell lines) were retrieved. Genotypes of 109 *IRF5 *polymorphisms, including four known functional polymorphisms, were considered. The best linear regression models accounting for the *IRF5 *expression data were selected by using a forward entry procedure. SLE-associated *IRF5 *haplotypes were correlated with the expression data and with the best *cis*-regulatory models.

**Results:**

A large fraction of variability in *IRF5 *expression was accounted for by linear regression models with *IRF5 *polymorphisms, but at a different level in each expression data set. Also, the best models from each expression data set were different, although there was overlap between them. The SNP introducing an early polyadenylation signal, rs10954213, was included in the best models for two of the expression data sets and in good models for the other two data sets. The SLE risk haplotype was associated with high *IRF5 *expression in the four expression data sets. However, there was also a trend towards high *IRF5 *expression with some protective and neutral haplotypes, and the protective haplotypes were not associated with *IRF5 *expression. As a consequence, correlation between the *cis*-regulatory best models and SLE-associated haplotypes, regarding either the risk or protective component, was poor.

**Conclusions:**

Our analysis indicates that although the SLE risk haplotype of *IRF5 *is associated with high expression of the gene, *cis*-regulation of *IRF5 *expression is not enough to fully account for *IRF5 *association with SLE susceptibility, which indicates the need to identify additional functional changes in this gene.

## Introduction

Systemic lupus erythematosus (SLE) [1-4], Sjögren's syndrome [5-7], systemic sclerosis [8-11] and primary biliary cirrhosis [12,13] are complex autoimmune diseases with a genetic component that includes among their strongest susceptibility loci the interferon regulatory factor 5 gene (*IRF5*). There are reports indicating that this gene can be associated with a subgroup of patients with rheumatoid arthritis [14-16] and patients with other autoimmune diseases [17-19]. The *IRF5 *gene encodes a transcription factor involved in the innate immune response as part of the type I IFN pathway, and its risk alleles have been associated with increased expression of this pathway [20,21]. Multiple polymorphisms in *IRF5 *are associated with disease susceptibility, but it is unclear which of them is causal and how these polymorphisms contribute to disease predisposition. This uncertainty is a serious obstacle to progress in these complex diseases.

Four polymorphisms with a putative functional role have been described. One of them is an insertion-deletion polymorphism (indel) changing 10 amino acids in exon 6, but experimental evidence of any effect associated with this indel is still lacking [22,23]. The other three polymorphisms are involved in processes that could influence expression levels of *IRF5*. The T allele of rs2004640 introduces a donor splice site that exchanges alternative first exons. It could affect levels of *IRF5 *mRNA through differences in *cis*-regulation [2], but its relevance has been questioned [22]. The CGGGG indel modulates binding of the Sp1 transcription factor in the *IRF5 *promoter [24], but it did not contribute independently to *IRF5 *levels in a study involving blood cells from healthy controls [25]. The strongest evidence of a role in *cis*-regulation has been found for the remaining functional polymorphism, rs10954213. Its A allele creates an early polyadenylation site that leads to a shorter mRNA isoform with an extended half-life and higher *IRF5 *expression in both lymphoblastoid cell lines (LCLs) [3,23] and blood cells [25]. However, according to studies done with LCLs, this SNP is not enough to fully account for *IRF5 cis*-regulation [3,23]. In addition, researchers in a study analysing *IRF5 *expression in blood cells from SLE patients did not find any significant effect of this SNP or of any of the functional polymorphisms [26]. These contrasting pieces of evidence do not allow for a clear understanding of *IRF5 cis*-regulation and its relationship to disease susceptibility.

*IRF5*-dependent disease susceptibility is determined by haplotypes with opposed effects: risk and protection [3-5,11,15,16,22,23]. The risk haplotype, identified by the rare allele of rs10488631 (or rs2070197), could be due to a combination of effects of the known functional polymorphisms, but its components are unclear. It has been proposed to result from the combination of two functional polymorphisms, rs2004640 and rs10954213, and a SNP of unknown relevance [3], or from a gradation of the effects of three functional polymorphisms, the two mentioned plus the exon 6 indel [23], or from an epistatic interaction between a unique combination of alleles at the same three functional polymorphisms [4]. Other studies have left this matter more or less undefined because of the lack of convincing evidence of the relevance of all the polymorphisms' segregating with the risk haplotype [22], or they have proposed, after the discovery of the CGGGG indel, that this functional polymorphism determines SLE increased risk together with not yet known functional polymorphisms [24]. The protective haplotypes are represented by the rare allele of rs729302 that is 5' to the gene, but are not correlated with any of the functional polymorphisms [3,4,23]. Therefore, none of the two effects has a clear relationship to known functional polymorphisms or to *IRF5 *function.

Here we address these questions using, for the first time, information from multiple mRNA expression studies and from the four known functional *IRF5 *polymorphisms. This approach allowed us to assess the reproducibility and generality of the results. Also, it afforded us the opportunity to test the independent contribution of each functional polymorphism and to identify the SNP introducing an early polyadenylation signal, rs10954213, as the clearest *cis*-regulatory one. In addition, we have confirmed that the SLE risk haplotype is associated with high *IRF5 *expression. However, the lack of correlations between *cis*-regulatory polymorphisms and SLE association and between *IRF5 *expression and SLE protective haplotypes indicates that SLE association involves changes in *IRF5 *function apart from its expression.

## Materials and methods

### Lymphoblastoid cell line expression data

**Figure 1:**
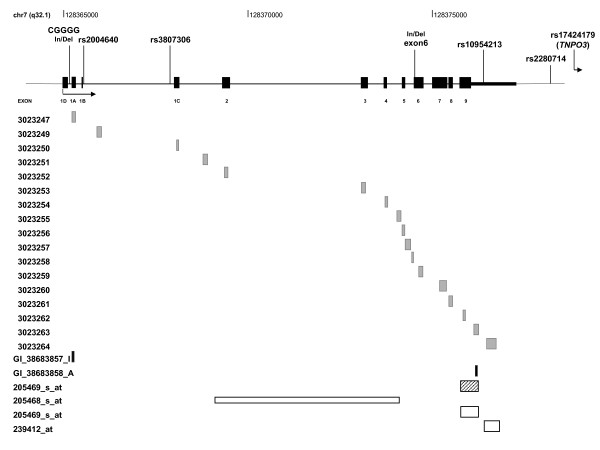
**Map of *IRF5 *locus**. Positions of relevant polymorphisms and of the probes included in each of the four microarray studies are indicated. Probes are colour-coded according to their reference source: light grey, Kwan *et al*. [29]; black, Stranger *et al*. [28]; striped pattern, Cheung *et al*. [27]; white, Dixon *et al*. [30].

*IRF5 *expression data from two collections of LCLs were obtained from four published microarray studies (Table 1). Three of the studies were done with LCLs from unrelated subjects derived from the European population from the International HapMap Project (CEU) [27-29], which lacks significant admixture and has been used as the reference for the European Caucasian population in many studies. The fourth study was done with LCLs from children with asthma [30]. That study included 206 UK families with negligible population stratification. We have used only the LCLs from each family having the best genotype call rate, leaving us with a total of 181. Data were obtained from the Gene Expression Omnibus repository [27,28] (accession numbers GSE6536 and GSE2552) or from the study authors [29,30]. Each study used a different microarray that included a variety of probes to examine *IRF5 *expression (Figure 1). We used expression data only from validated probes in each study.

**Table 1:** Expression profiling studies in lymphoblastoid cell lines whose *IRF5 *data have been analysed^a^

Study	Number of LCLs	Microarray system used	Number of *IRF5 *probes used	LCL collection group
Kwan *et al*. [29]	57	GeneChip Human Exon 1.0 ST Array (Affymetrix, Inc.)	17	CEU
Stranger *et al*. [28]	60	Illumina WG-6v1 BeadChip Array (Illumina, Inc.)	2	CEU
Cheung *et al*. [27]	58	GeneChip Human Genome Focus Array (Affymetrix, Inc.)	1	CEU
Dixon *et al*. [30]	181^b^	GeneChip Human Genome U133 Plus 2.0 Array (Affymetrix, Inc.)	3	Asthma

^a^*IRF5 *= interferon regulatory factor 5 gene; LCL = lymphoblastoid cell line; CEU = European population from International HapMap Project database; ^b^number of LCLs selected for having the best genotyping call rate per family among the 400 LCLs available.

### *IRF5 *genotypes of the lymphoblastoid cell line

The linkage disequilibrium (LD) block encompassing *IRF5 *was defined according to International HapMap Project data on the CEU population between 128,158 kb and 128,304 kb on chromosome 7 (HapMap Rel 21a/phase II Jan07, NCBI B35, dbSNPb125). Genotypes of the CEU LCLs for the 72 SNPs included in this 146-kb region (Additional file 1, Table S1) were downloaded from the International HapMap Project (HapMap) [31]. Data from 27 SNPs in this LD block were available in the asthma collection of LCLs.

### Genotyping and imputation of additional genotypes

We obtained complete genotype information in the *IRF5 *LD block to a total of 109 polymorphisms (Additional file 1, Table S1) by imputation using MACH 1.0 software [32]. Information for imputation was taken from three sources: the 72 SNPs that had been studied in HapMap LCLs, the 27 SNPs available in the asthma LCLs and the 56 SNPs that we have genotyped in 95 healthy Spanish donors. In this way, it was possible to include the four putative functional polymorphisms (absent from HapMap) and more SNPs in the 5' region of *IRF5 *(seven SNPs in tight LD with rs729302, the tagging SNP for the protector haplotypes in SLE) (Additional file 2, Figures S1 and S2). There were overlaps between the HapMap and asthma data sets (21 SNPs) and between HapMap and the Spanish donors (nine tagging SNPs). Genotypes of the Spanish donors were obtained by using the ABI PRISM SNaPshot Multiplex Kit (Applied Biosystems, Carlsbad, CA, USA) as described previously [4], except for rs3778752, rs3778751 and the CGGGG indel, which were sequenced (Additional file 1, Tables S2 and S3), and the exon 6 indel that was genotyped by length variation in agarose electrophoresis as described previously [4]. Polymorphisms with a MACH 1.0 quality score < 0.8 were discarded.

DNA samples from controls were obtained with their informed written consent, and the study was approved by the Committee for Clinical Research of Galicia (Spain).

### Statistical analysis

Expression results from probes targeting introns were excluded from the analysis. Expression data were transformed into standardised normal distributions (that is, expression data from each probe were transformed into new variables with mean = 0 and standard deviation = 1 by subtracting the mean to each value and dividing the result by the standard deviation) to avoid differences in scale when performing comparisons between studies. Relations of the expression results with the *IRF5 *polymorphisms were analysed by multiple linear regression. These analyses were performed with a forward entry procedure, which adds new polymorphisms to the regression model one-by-one, starting with the most associated until no further significant improvement is achieved or until one of the polymorphisms does not show a significant contribution to the model. A genetic additive model (with values 0, 1 and 2 for the AA, Aa and aa genotypes, respectively) was considered. Only one of each pair of polymorphisms, to a total of 35 polymorphisms, with *r*^2 ^≥ 0.90 was included in the analyses to avoid collinearity problems (Additional file 1, Table S1). Nested linear regression models were compared using the likelihood ratio test. Nonnested models were compared using Davidson and MacKinnon's J-test [33], which specifies a proxy parameter in an artificial nested model combining the two nonnested models and then tests the proxy parameter. All analyses were done using Statistica 7.0 software (StatSoft, Tulsa, OK, USA) or in R software implementations, except for haplotype estimation, which was done using Phase 2 software [34].

## Results

### *IRF5 *expression in lymphoblastoid cell lines

**Figure 2:**
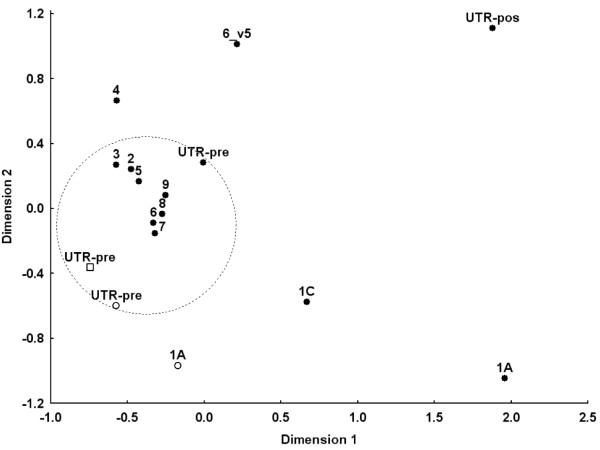
**Correlation between *IRF5 *expression results obtained with different probes and in different experiments**. Only results obtained with the same European population from the International HapMap Project (CEU) lymphoblastoid cell line (LCL) are compared. Bidimensional, nonparametric, multidimensional scaling was used for representation. Data from probes selected as representative are within the dashed circle. Labels for data from each probe indicate the number of the exon they target, including the 1A and 1C alternative exons and a variant sequence of exon 6 (6_v5), and UTR-pre and UTR-pro for the probes targeting the 3'UTR previous or posterior to rs10954213, respectively. Filled circles correspond to data from Kwan *et al*. [29], empty circles correspond to data from Stranger *et al*. [28] and empty squares correspond to data from Cheung *et al*. [27].

To ascertain *cis*-regulatory *IRF5 *polymorphisms, we selected four studies (Table 1) containing *IRF5 *genotypes and microarray expression data in LCLs [27-30]. The multiplicity of studies and hybridisation probes (Figure 1) allowed us to select the most representative expression results. As a first step in this process, we used the study by Kwan *et al*. [29], which included 13 probes targeting specific *IRF5 *exons in LCLs from the CEU population of HapMap. Two different groups of results were identified (Figure 2). The first group included eight probes that were highly correlated (mean pairwise *r*^2 ^= 0.79). They hybridised with exons 2, 3, 5, 6 (not including the 30-bp indel), 7, 8 and 9 and with the 3'UTR previous to the early polyadenylation signal SNP rs10954213. The uncorrelated results were obtained with probes hybridising with exons 1A and 1C, which are alternatively spliced and untranslated; with exon 4, which is the smallest; and with the sequence of exon 6, which is present only in splice variant 5. We took the average of the first group as representative of *IRF5 *expression and named it K8.

The other two microarray studies done with CEU LCLs contained fewer *IRF5 *probes. The Stranger *et al*. study [28] included probes hybridising with exon 1A and with the 3'UTR previous to rs10954213 (Figure 1). Only results from the second probe correlated with K8 (*r*^2 ^= 0.56), and they were taken as representative and called S (Figure 2). The Cheung *et al*. study [27] included only one *IRF5 *probe (Figure 1), which hybridised with the upstream region of exon 9 and the 3'UTR. The results of this probe, which we identified as C, strongly correlated with the results for K8 (*r*^2 ^= 0.60) and S (*r*^2 ^= 0.75) (Figure 2). The high correlation between the three data sets, K8, S and C, permitted us to obtain a global average that was denoted KSC. For an analysis of the unselected data, see Additional file 2, Supplementary Information.

To increase the generality of the results, we used a fourth study that had examined a different collection of LCLs [30]. That study included data derived from three *IRF5 *probes (Figure 1), which were poorly correlated (not shown). We considered as representative only the probe that was shared with the study by Cheung *et al*. [27] and targeted sequences addressed in the other two studies. These data were named D.

### Best genetic models of *IRF5 cis*-regulation

The forward entry multiple linear regression process led to the identification of four best models, one for each of the data sets and a best model for the KSC average that included the same polymorphisms as the best model for S. The best model for the K8 results, which included genotypes at two SNPs, rs3807306 and rs17424179 (Table 2), explained 0.31 of the variance in *IRF5 *expression. The first SNP, rs3807306, is in *IRF5 *intron 1 and is the most associated in the model. This SNP showed an *r*^2 ^value larger than 0.7 with 22 polymorphisms, including two functional ones: rs10954213 (*r*^2 ^= 0.79) and the CGGGG indel (*r*^2 ^= 0.75). The second SNP, rs17424179, is 68 kb 3' to *IRF5*. It did not show a strong correlation with any other polymorphism (all pairwise *r*^2 ^< 0.2). Its contribution to the model fit was small. The best model for S results included three SNPs that accounted for a very large fraction of variability in *IRF5 *expression (adjusted *r*^2 ^= 0.80). The strongest association in this model was with rs10954213 (Table 2). The two other SNPs were the same as the best model for K8, rs3807306 and rs17424179. The best model for the C expression data also accounted for a large fraction of variability (adjusted *r*^2 ^= 0.55), including only two SNPs (Table 2). The major contribution corresponded to rs2280714, which is 4.6 kb 3' to *IRF5 *and showed a strong correlation with the second SNP in the model, rs10954213 (*r*^2 ^= 0.84), and with 25 other SNPs (*r*^2 ^> 0.7). As this model includes two highly correlated SNPs, their independent contributions were severely reduced in relation to the model fit (*P *= 0.02 for each of the two SNPs in a model with *P *= 9.2 × 10^-11^). As a form of summary of these three data sets, the average KSC expression results were analysed. They were well accounted for (66% of the variability) by a best model with three SNPs that were the same and in the same order as those in the best model for S data (Table 2). Therefore, the three studies with the same cell lines showed expression data that could be largely explained by *cis*-regulation because of a small number of polymorphisms.

**Table 2:** Best multiple linear regression models with *cis*-polymorphisms accounting for *IRF5 *gene expression in each of the four data sets and in the average expression from CEU LCL (KSC)^a^

	Best linear regression model			Polymorphism *P *value in model
	Data set	Adjusted *r*^2^	Model *P*	Polymorphisms
K8 (Kwan *et al*. [29])	0.31	1.7 × 10^-5^	rs3807306	4.0 × 10^-6^
			rs17424179	0.026
S (Stranger *et al*. [28])	0.80	2.5 × 10^-20^	rs10954213	1.2 × 10^-4^
			rs3807306	2.4 × 10^-3^
			rs17424179	9.5 × 10^-3^
				
C (Cheung *et al*. [27])	0.55	9.2 × 10^-11^	rs2280714	0.02
			rs10954213	0.02
KSC	0.69	1.3 × 10^-13^	rs10954213	5.1 × 10^-3^
			rs3807306	0.013
			rs17424179	0.011
D (Dixon *et al*. [30])	0.28	1.3 × 10^-13^	CGGGG indel	2.4 × 10^-6^
			rs2280714	8.3 × 10^-4^

^a^*IRF5 *= interferon regulatory factor 5 gene; LCL = lymphoblastoid cell line; CEU = European population from International HapMap Project database; KSC = average of standard normal transformed K8, S and C data. Statistical parameters of the best models are provided together with *P *values corresponding to independent contribution of each polymorphism to the model. Data sets in left column are representative *IRF5 *expression results.

**Figure 3:**
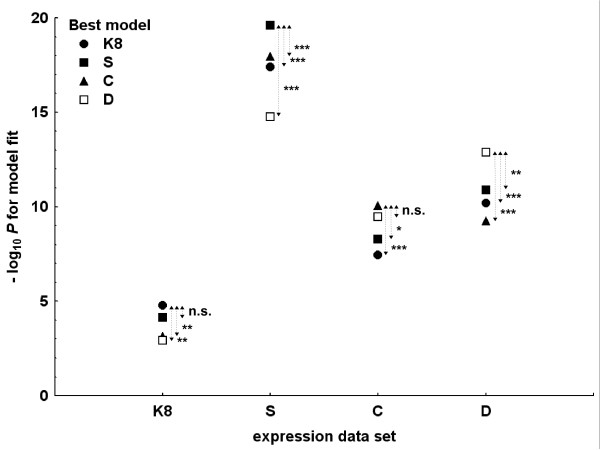
**Cross-check analysis of the best linear regression genetic models**. Polymorphism composition of each of the best models from Table 2 was applied to the four expression data sets and the -log_10 _*P *values for their fit are represented. Models defined as best in expression data sets with CEU LCL are shown in black (triangles for K8, squares for S and circles for C), and the best model defined with asthma cell lines in D is presented as white squares. Expression data sets labels in the X-axis are as in Table 2. Comparisons with the proper best model for each data set were either nonsignificantly inferior (n.s.) or inferior with *P < 0.05, **P < 0.01 or ***P < 0.001.

The best model for the independent D results comprised two polymorphisms: the functional CGGGG indel and the already mentioned rs2280714 (Table 2), which is strongly correlated with rs10954213, among others. The polymorphism composition of the best genetic models was applied to the other expression data sets to explore their relationships. The exchanged models were significantly inferior to the proper best models, with two exceptions (Figure 3): the model with the best combination of polymorphisms for the S data set was equivalent to the best model in the K8 data, which it contained; and the model with the best combination of polymorphisms for the D data set was equivalent to the best model in the C expression data. The SNP composition rs10954213, rs3807306 and rs17424179 produced the best model overall: best for the S data set, not significantly different from the best in the K8 data set, second best for the D data and third best for the C data set. In addition, it comprised the SNPs in the best model for the KSC average data.

To ascertain the origin of differences between data sets, we compared model fit with the scale of expression levels, the range of values and their dispersion, as well as with sample size differences, but no correlation was found. Also, we assessed the best model for K8 in the results from each of the eight exons in the study by Kwan *et al*. [35]. The fit of the model ranged from *r*^2 ^= 0.12 for data from exon 9 and 3'UTR (previous to rs10954213) to *r*^2 ^= 0.37 for data from exon 7 (*P *= 0.01, and 1.7 × 10^-6 ^in linear regression analyses, respectively). These two exons are shared by all known *IRF5 *isoforms. Therefore, these differences point to the probes as the source of variability because the results are from the same hybridisation experiment, cancelling all variation involved in cell culture, mRNA extraction and cDNA synthesis or labelling. In contrast, data sets C and D shared the same probe, but they also showed differences in model fit that should be ascribed, in this case, to other unidentified factors that could include laboratory procedures and the collection of cells from healthy subjects and asthma patients in C and D set, respectively.

### Role of the putative functional polymorphisms

**Figure 4:**
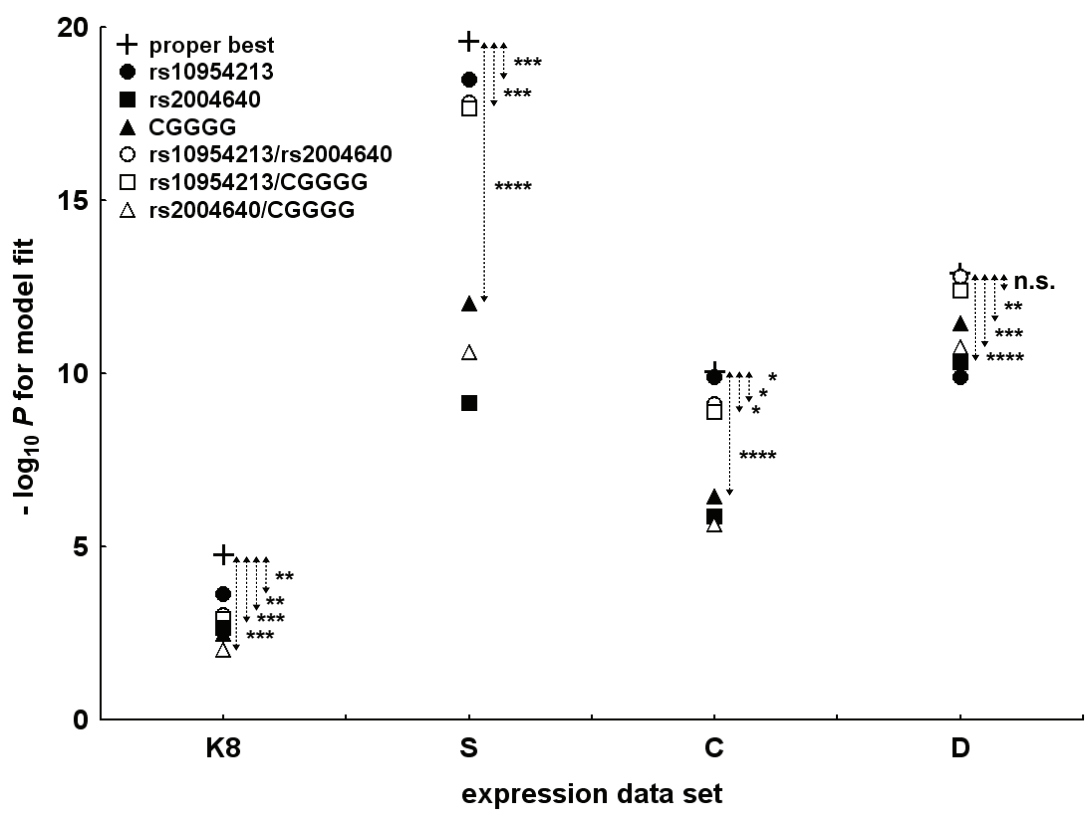
**Evaluation of linear regression models made only of known functional polymorphisms**. The -log_10 _*P *value for the fit of the models applied to each data set are represented in ordinates and compared with the proper best models (plus signs). Models including only one of the functional polymorphisms are indicated by filled symbols, and models combining two polymorphisms are shown as open symbols. Models included either rs10954213 (filled circles), rs2004640 (filled squares), the CGGGG insertion-deletion polymorphism (indel) (filled triangles), rs10954213 and rs2004640 (open circles), rs10954213 and the CGGGG indel (open squares) or the rs2004640 and the CGGGG indel (open triangles). Comparisons with the proper best model for each data set were either nonsignificantly inferior (n.s.) or inferior with *P < 0.05, **P < 0.01, ***P < 0.001 or ****P < 0.0001. Expression data sets in the X-axis are as in Table 2.

We have analysed how well models in which only functional polymorphisms were included accounted for the expression data (Figure 4). Models including the exon 6 indel were clearly inferior and are not shown, given the lack of any previous evidence of the involvement of the exon 6 indel in *IRF5 cis*-regulation. Each of the remaining three functional polymorphisms, considered individually, was significantly associated with *IRF5 *expression in the four data sets. The early polyadenylation signal SNP, rs10954213, was clearly dominant among the functional polymorphisms in these individual comparisons, except in the D data set. However, none of them alone was able to account for *IRF5 *expression equivalently to the proper best genetic model for each data set. Models combining functional polymorphisms were not better than models with rs10954213 alone in the three data sets with CEU LCLs (K8, S and C). In contrast, the two models including rs10954213, together with rs2004640 or with the CGGGG indel, were the best in accounting for the D expression data and were not inferior to the proper best model. It is important to note that in these two models and in this data set, the two component polymorphisms showed a significant independent contribution (Additional file 2, Table S1). In the other three data sets (K8, S and C), the best models with two functional polymorphisms included rs10954213 with rs2004640 and, immediately below, rs10954213 with the CGGGG indel. Only rs10954213, however, showed a significant independent contribution in these combined models (Additional file 2, Table S1).

A search for other putative functional polymorphisms in the *IRF5 *sequence with two bioinformatics applications, Pupasuite 3.1 [36] and FastSNP [37], gave only an SNP that could introduce an alternative splice site in intron 1, but it was not polymorphic in our 95 Spanish samples.

### Relationship between *IRF5 *expression and systemic lupus erythematosus susceptibility

**Figure 5:**
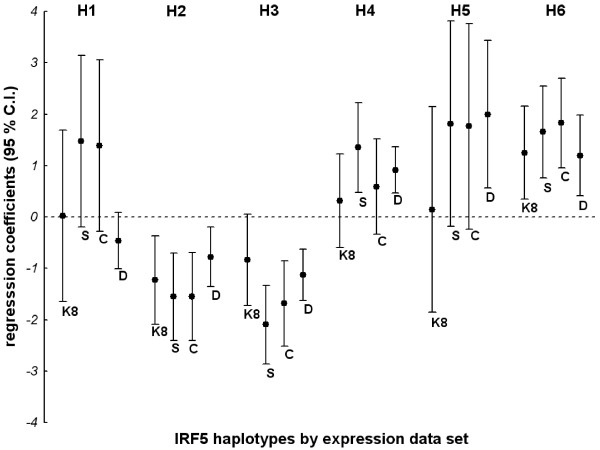
**Relationship between *IRF5 *haplotypes and expression in each of the expression data sets**. The most common haplotypes of the interferon regulatory factor 5 (*IRF5*) gene were defined with eight tag SNPs and from H1 to H6 as described by Ferreiro-Neira *et al*. [4] (for haplotype definition, see Supplementary Table 5). H1 and H2 are systemic lupus erythematosus (SLE) protective haplotypes, and H6 is the risk haplotype. The remaining haplotypes are neutral. Univariate linear regression coefficients with their 95% confidence intervals (95% C.I.) for the relationship between each haplotype (coded 0, 1 or 2 if absent, heterozygous or homozygous, respectively) and *IRF5 *expression are shown. Coefficients significantly larger than 0 (with C.I. not crossing the dashed line at 0) indicate an association with increased *IRF5 *expression. Coefficients significantly smaller than 0 show association with decreased *IRF5 *expression. The values have been rescaled to allow the use of a single *y*-axis. Codes for each of the *IRF5 *expression data sets are given in Table 2.

We used haplotypes defined in previous reports to assess the relationship between *IRF5 *expression and SLE susceptibility [2,4] (Additional file 2, Table S2). The SLE risk haplotype H6 is identified by the minor allele of rs10488631. The protective haplotypes H1 and H2 are defined by the minor allele of rs729302, with H1 including the A allele of rs10954213 and H2 including the G allele. They share the minor allele of rs2004640 with the neutral haplotype H3, but this latter haplotype lacks the minor allele of rs729302. H4 and H5, which are SLE-neutral, are very similar to the risk haplotype H6 but lack the minor allele of rs10488631. None of the best models for any of the expression data sets was strongly correlated (all *r*^2 ^≤ 0.15) with the haplotypes defining SLE risk (H6) or SLE protection (H1 and H2) (Additional file 2, Table S3). However, we found that the minor allele of rs10488631 that identifies the SLE risk haplotype was significantly associated with increased *IRF5 *expression in all data sets (all *P *< 0.0094). On the contrary, the minor allele of rs729302 that identifies the SLE protection haplotype was associated with lower expression of *IRF5 *only in the D data set (*P *= 0.002), but not in the other data sets (not shown). In addition, analysis of the association of the estimated haplotypes showed that the only haplotype consistently associated with high *IRF5 *expression in all data sets was the risk haplotype H6 (Figure 5). However, this finding was not specific, because there was an association of higher *IRF5 *expression with neutral haplotypes H4 and H5 in some data sets. There was also poor correlation between SLE-protective haplotypes and *IRF5 *expression. Of the two protective haplotypes, only the H2 haplotype was consistently associated with lower *IRF5 *expression (all *P *values within the range from 0.009 to 0.0008) (Figure 5). The H1 haplotype was not associated with *IRF5 *expression in any of the four data sets (all *P *> 0.09).

## Discussion

Identification of SLE causal polymorphisms in *IRF5 *is very difficult. A thorough analysis with novel characteristics including the use of expression data from four different studies, the inclusion of genotypes of the four known functional polymorphisms, and the direct assessment of the relationship between SLE-associated haplotypes and *IRF5 *expression has provided new and interesting insights.

The multiplicity of probes and studies allowed us to select the most representative *IRF5 *expression data. They included almost all the probes for *IRF5 *transcribed sequences that are common to all isoforms. They showed good correlation (*r*^2 ^≥ 0.56) between the three experiments using the same LCLs. However, differences between experiments led to consequences in the results, such as with regard to the fraction of expression variability accounted for by the best *cis*-regulatory models, which ranged from 0.28 to 0.80. There is no simple cause of these differences. They did not correlate with sample size, with the scale of the expression values or with their dispersion. However, there were notable differences within the same experiment that were dependent on the hybridisation probes. Other undefined factors, which could include cell culture, sample processing and differences between cell collections, were also suggested by our analyses.

Differences between the experiments were also evident in the best *cis*-regulatory models. Each expression data set was best explained by a specific genetic model, but the polymorphisms included in them were partially coincident. The best assessment of the relationships between the four best genetic models was obtained by applying the models to the other data sets as a sort of cross-checking procedure (Figure 3). This analysis showed that it was impossible to identify a single best model explaining *IRF5 *expression, but it was possible to define a range of best *cis*-regulatory models that were useful for assessing hypotheses.

The multiplicity of best *cis*-regulatory models makes it uninteresting to comment on the implications of each. However, there was a specific combination of SNPs worth discussing because it was superior to the others in cross-comparisons. It contained rs10954213 (see next paragraph) together with rs3807306 and rs17424179 (Figure 3). There has been no previous specific mention of rs17424179, but rs3807306 has been found to account for most haplotype effects in SLE association among African Americans [38], and it has been highlighted among the *IRF5 *polymorphisms particularly associated with multiple sclerosis [18] and rheumatoid arthritis [14]. In addition, a model with rs3807306 and rs10954213 was the second best to account for SLE association in a study in Caucasians [24]. Some of these associations have been interpreted as if this SNP acted as a proxy for the CGGGG indel because of LD between them and lack of an allele-specific effect of rs3807306 in an electrophoretic mobility shift assay [18,24]. However, this interpretation is not applicable to our results, because the indel was also included in the analyses. Therefore, it is likely that rs3807306 indicates additional functional polymorphisms or a combination thereof. Its role in *IRF5 cis*-regulation is also supported by the analysis of Rullo *et al*. [21], in which this SNP showed the strongest association with *IRF5 *levels among the 14 SNPs considered, in both the European and the Asian collections of HapMap LCLs.

The SNP determining early *IRF5 *polyadenylation, rs10954213, was clearly dominant in accounting for the expression data obtained with CEU LCLs (Table 2). This predominance of rs10954213 in the CEU LCLs was confirmed in the analyses limited to the known functional polymorphisms (Figure 4). Overall, our analyses provide strong evidence supporting the role of rs10954213 in *cis*-regulating *IRF5 *expression. They are compatible with its identification as the main *IRF5 cis*-regulatory polymorphism in blood cells from healthy controls [25] and with previous studies in which its role and mechanism of action were elucidated [2,3]. However, Feng *et al*. [26] recently reported a lack of association, a result that could be due to insufficient power because only 14 to 26 subjects were considered in these specific comparisons.

The dominant role of rs10954213 in the CEU LCL data was such that all the genetic models with known functional polymorphisms were inferior to the model including only rs10954213. These results suggest that the other three known functional polymorphisms are redundant in *IRF5 *expression. This conclusion should be tempered by the discordant results obtained with the asthma LCL expression data. They showed a significant contribution to the best functional *cis*-regulatory models from either rs2004640 or the CGGGG indel in combination with rs10954213. However, we do not know which of the results with the two LCL collections is more representative of the population at large.

We found a consistent association of the SLE risk haplotype with higher *IRF5 *expression in the four data sets (Figure 5). This association has already been demonstrated in SLE and healthy control blood cells [25,26]. It has been the focus of attention in previous reports and is the basis of the hypothesis that *IRF5 *risk alleles act by potentiating the type I IFN pathway. This hypothesis has received recent experimental support in studies done with SLE sera [20] and with LCLs [21].

Our results also indicate that there is more than *IRF5 *expression in *IRF5*-dependent disease association. This was shown by the lack of correlation of the SLE susceptibility haplotypes with the best *cis*-regulatory models and with *IRF5 *expression in either the SLE risk or SLE protective haplotype. We do not yet have a good hypothesis of what the additional changes in *IRF5*, besides its expression, could be. Possibilities include alteration of interactions with other proteins, as has been suggested for the exon 6 indel [22,23], or changes in the isoforms by alterations in splicing, a mechanism demonstrated for rs2004640 [2], but with little relevance [22]. A search for other putative functional polymorphisms using bioinformatics tools did not lead us to new hypotheses. Therefore, the need to continue studying *IRF5 *polymorphisms to understand their role in disease susceptibility is an imperative.

One of the limitations of our study is that only global *IRF5 *expression data, as opposed to isoform-specific data, were obtained from LCLs in basal conditions, which could be different from the relevant *IRF5 *isoform, cell type or activation status. However, it is important to note that no significant *cis*-regulation for *IRF5 *isoforms has yet been reported, in spite of its many splice variants and their upregulation in SLE [22,26]. In addition, results with blood cells have been concordant with results with LCLs [25], and the *IRF5 *risk haplotype has also been found to be associated with overexpression of *IRF5 *in the blood cells, monocytes and myeloid dendritic cells of SLE patients [26]. An additional limitation which we acknowledge is the possibility that some of the best models could be different with the use of actual genotypes in place of imputed ones. Finally, our study included only LCLs from European Caucasians. This was done on purpose because there are differences in the structure of *IRF5 *haplotypes and their SLE associations and differences in *IRF5 cis*-regulation between Europeans, Asians and Africans [21,38,39].

## Conclusions

Our study has shown significant variability in results from different studies of *IRF5 cis*-regulatory polymorphisms. However, this variability is compatible with the finding that *cis*-regulatory changes in *IRF5 *expression are not sufficient to explain their association with SLE, although there is a consistent association of the SLE risk haplotype with high *IRF5 *expression.

## Abbreviations

CEU: European population from the International HapMap Project; CI: confidence interval; IFN: interferon; *IRF5*: interferon regulatory factor 5 gene; LCL: lymphoblastoid cell line; OR: odds ratio; SLE: systemic lupus erythematosus; SNP: single-nucleotide polymorphism; UTR: untranslated region.

## Competing interests

The authors declare that they have no competing interests.

## Authors' contributions

EAP genotyped the samples and participated in the interpretation of the results and the writing of the manuscript. MSG participated in the design of the study, obtained genotype data and participated in the interpretation of the results and the writing of the manuscript. MC participated in the design of the study, in statistical analysis and in the interpretation of the results. TK and JM provided detailed microarray data and participated in the interpretation of the results and the writing of the manuscript. JJGR participated in the analysis and interpretation of the results. AG participated in the design of the study and the acquisition of data and supervised the genotyping, statistical analysis, interpretation of results and the writing of the manuscript. All authors read and approved the final manuscript.

## Supplementary Material

Additional file 1 **Supplementary materials and methods**. Interferon regulatory factor 5 (*IRF5*) gene polymorphisms that have been studied, with indications of the sources of their expression data as well as the primers and probes that were used to genotype them. Click here for file

Additional file 2 **Supplementary results**. Complementary analyses of the *IRF5 *lineal regression models and of the haplotype distribution, together with linkage disequilibrium maps and expression results pertaining to probes targeting less representative *IRF5 *exons. Click here for file
