# Supplementary material for: Cis-regulation of IRF5 expression is unable to fully account for systemic lupus erythematosus association: analysis of multiple experiments with lymphoblastoid cell lines
Source: Arthritis Res Ther. 2011 May 31;13(3):R80. doi: 10.1186/ar3343 (PMC3218890; doi:10.1186/ar3343)
Supplement: Additional file 1 — Supplementary materials and methods. Interferon regulatory factor 5 (IRF5) gene polymorphisms that have been studied, with indications of the sources of their expression data as well as the primers and probes that were used to genotype them. [file ar3343-S1.DOC]

**Supplementary Table 1**: List of the 109 polymorphisms analyzed in the IRF5 LD block. Polymorphism names in bold indicate the 35 polymorphisms that were selected for the analyses of correlation with IRF5 expression because they were not redundant at a pairwise r^2^ = 0.90. The + sign means that genotypes for these polymorphisms were available in the indicated LCL collection (CEU = European HapMap; Asthma = children with asthma from Dixon et al. [30]) or in the healthy Spanish controls used to complete the LD map (Controls)

| Polymorphism | CEU | Asthma | Controls |
| --- | --- | --- | --- |
| **rs4731528** | - | - | + |
| **rs10081379** | - | - | + |
| **rs10280295** | - | - | + |
| rs6970780 | - | - | + |
| **rs6951243** | - | - | + |
| **rs960633** | - | - | + |
| **rs4731530** | - | - | + |
| rs4731531 | - | - | + |
| **rs2402941** | - | - | + |
| **rs6968225** | - | - | + |
| rs6968508 | - | - | + |
| rs6950728 | - | - | + |
| rs11982901 | - | - | + |
| rs4728141 | - | - | + |
| rs13245639 | - | - | + |
| **rs729302** | + | + | + |
| rs729068 | - | - | + |
| rs12706860 | - | - | + |
| rs7808659 | - | - | + |
| rs2402940 | + | - | + |
| rs754284 | - | - | + |
| rs754281 | - | - | + |
| rs11768806 | - | - | + |
| **rs4728142** | + | + | + |
| rs7801838 | - | - | + |
| **rs1874330** | - | - | + |
| rs3778754 | - | - | + |
| rs3757388 | - | - | + |
| rs3757387 | - | - | + |
| rs3757385 | - | - | + |
| **rs3807134** | - | - | + |
| **rs3807135** | - | - | + |
| **CGGGG indel** | - | - | + |
| **rs2004640** | - | - | + |
| rs3807307 | - | - | + |
| rs752637 | + | - | + |
| rs3823536 | - | - | + |
| rs3778753 | - | - | + |
| **rs3807306** | + | - | - |
| **rs11761199** | + | - | - |
| **rs7808907** | + | + | - |
| **rs1874328** | + | + | - |
| **In/Del Exon6** | - | - | + |
| **rs10954213** | - | - | + |
| rs13242262 | + | - | + |
| **rs10488630** | + | + | + |
| **rs10488631** | + | + | + |
| **rs2280714** | + | + | + |
| rs10236569 | + | - | - |
| **rs6966125** | + | - | - |
| rs10229001 | + | - | - |
| **rs1495458** | + | - | - |
| rs2172876 | + | - | - |
| **rs6957529** | + | - | - |
| rs7385716 | + | - | - |
| rs4731535 | + | - | + |
| rs8043 | + | - | - |
| rs1874332 | + | + | - |
| rs2293492 | + | + | - |
| rs12531711 | + | - | - |
| rs17338998 | + | - | - |
| rs2272347 | + | - | - |
| **rs3817555** | + | - | - |
| rs7789423 | + | + | - |
| rs6948928 | + | + | - |
| **rs12534421** | + | - | - |
| rs12535158 | + | - | - |
| rs12669885 | + | - | - |
| **rs1154330** | + | + | - |
| rs17339221 | + | - | - |
| rs2290231 | + | - | - |
| rs11770317 | + | - | - |
| rs1154329 | + | - | - |
| rs6969930 | + | - | - |
| rs2305323 | + | - | - |
| rs3958094 | + | - | - |
| rs7807018 | + | - | - |
| **rs2305324** | + | + | - |
| rs11768572 | + | - | - |
| rs2305325 | + | + | - |
| rs6965542 | + | - | - |
| rs3847099 | + | - | - |
| rs3857852 | + | + | - |
| rs12539476 | + | - | - |
| **rs17424179** | + | - | - |
| rs12155080 | + | + | - |
| rs13236009 | + | - | - |
| rs13221560 | + | - | - |
| rs10239340 | + | + | - |
| rs11762968 | + | - | - |
| rs9649520 | + | - | - |
| rs1072767 | + | - | - |
| rs921403 | + | - | - |
| rs4731541 | + | - | - |
| **rs3993439** | + | - | - |
| **rs1839600** | + | - | - |
| rs3807301 | + | - | - |
| rs10279821 | + | + | - |
| rs10156169 | + | - | - |
| rs11767238 | + | - | - |
| rs17424602 | + | - | - |
| rs2167273 | + | + | - |
| **rs6960994** | + | + | - |
| rs6961014 | + | + | - |
| rs6980198 | + | - | - |
| rs2242028 | + | - | - |
| rs13239597 | + | - | - |
| rs11767954 | + | - | - |
| rs13246321 | + | - | - |

**Supplementary Table 2:** Primers and probes that were used for genotyping the *IRF5* polymorphisms in the healthy Spanish controls by minisequencing. Design of oligonucleotides and protocol of genotyping have already been described (Ferreiro-Neira et al.[4]). Some probes ($) were extended with a 5' tail (in capitals) that has no homology with human sequences. Others were mutated (underlined capitals) to avoid dimer formation.

| **SNPs** | **Oligonucleotides** |
| --- | --- |
| rs4731530 C>T | Forward cccctcacagtccagtagga  Reverse gcccgtggaagtaaattgtc  Probe atatttagacattgaaggccccagttct |
| rs4731531 G>A | Forward taacagatccgcaatggcta  Reverse tttcctggggaccatcatag  Probe TTACCgagcttggtgcctcacaactctaggggg$ |
| rs10280295 C>T | Forward ctcaccactccattgcttcc  Reverse tgccaagatcatatggcaag  Probe gaggatttctggcaatgtccagtgctcattgat |
| rs6951615 C>T | Forward ggacactgctggttccctta  Reverse ggcatgaaagcacagagtga  Probe TTACCTATGATTGATCGTGGTGATATcgcctcagcatcatatgTtgctactgc$ |
| rs6968508 C>G | Forward ccaatgcaagggggtagtta  Reverse ccactatgcccagcctaaact  Probe tggtatgtgacttatatctcaataaagctgttacaaaaaaggggagcg |
| rs10081379 T>A | Forward acactgacctccagccaagt  Reverse cagcaatattcaggggctct  Probe TTACCTATGATTGATtactaggtgttctccctagaaatgttatcgtcatgagaagtcc$ |
| rs12536195 G>A | Forward acctgtctgcgatgctcct  Reverse gctatctgtctggcgtctcc  Probe TTACCTATGAccggtaagtgccagcctcctcgagtggt$ |
| rs11773414 G>A | Forward gtggtgcaatcacagctcac  Reverse accgtcctggacaacatagg  Probe tcatgcctggctagttttctttattttttaattttgtagagac |
| rs6955705 T>C | Forward acctcagtctgctcccacag  Reverse tcaggagtgagtgggtaggc  Probe TTAcagcctcagccccatctcctgcatg$ |
| rs6970780 T>C | Forward catctccctctgaggtccaa  Reverse ccctgtctcattcaccactg  Probe agtgccttgcaaataacaccctttcctc |
| rs6950728 G>A | Forward tggctctgatgttagttgcat  Reverse agaaaactggtggggctgta  Probe TTACCTATGAtatgtttataattatcacatcttcctgatggattacacttttatcatt$ |
| rs4731528 C>T | Forward acctcagtctgctcccacag  Reverse tcaggagtgagtgggtaggc  Probe TTACCTATGATTGAgcctgccacctgagagtcagcgag$ |
| rs6951243 G>A | Forward caaggcacttgagttccaca  Reverse gtgctcatgtggttcccttt  Probe TTACCTATGATTGATCGTGGgttctgggcaggaccctgtctcattcac$ |
| rs6968225 C>G | Forward gacaacagcctgtcctcaca  Reverse ggcctctaactacccccttg  Probe TTACCTATGATTGATCGTGGTGATAccataagtcccaccccactgtttagagg$ |
| rs11768806 C >T | Forward gaggcttgagatggatctgg  Reverse cctccggtatgcacttttgt  Probe TTACCTATGATTGATCGTGGggtggaTctcgtgggcctggctgggaca$ |
| rs3757388 A>G | Forward aaaaaggttcccattttgtgg  Reverse gctaaggcaggcagatcact  Probe TTACCTATGATTGATCGTGGTGATAttagcctggcgtgTtgacacacacctatagttc$ |
| rs2402941 G>A | Forward tgtacctgccccataccttc  Reverse gtgtgcccatccaaataacc  Probe TTACCTATGATTGATCGTGGTGcaagtggcttctcggcacactgagaa$ |
| rs754280 G>A | Forward cggtggagatgttcctgaat  Reverse gggggcaggttcttactagg  Probe TTACCTATGActggcttggagaattgcaaagcaccaaggctcc$ |
| rs3757387 T>C | Forward gcctcccaagtaggtggaac  Reverse atggatggggaaaatgtgaa  Probe ccacgccaggctaatttttgtattttttgtagagacaaggttt |
| rs7801838 C>T | Forward aaagctctgagccggtgtta  Reverse gctttgaagtttctggcaca  Probe TTACCTATGATTGATggattaatgcccagggcgccacagctgg$ |
| rs3807134 T>C | Forward gaagctatttgcaccctgga  Reverse tctgaaccgttttcgattcc  Probe TTACCTATGATTGATCGTGGgagaaggaacaggaggtgtgtgaaggtggaggt$ |
| rs3807135 C>T | Forward cacatctggaaggggtgtct  Reverse tagactggccactggctctt  Probe TTACCTATGATTGATCGTGGTGActccagTccctcctctcgcctgcct$ |
| rs41298401 C>G | Forward gcgggatgaagactggagta  Reverse ggaggcgctttggaagtc  Probe TTACCTATGATTGATCGTccgTgcgcaccctgctgtag$ |
| rs3807307 C>T | Forward cccagaatggggataagtga  Reverse ccccaaagtagggcctttag  Probe TTACCTATGATTGATCGTGGTagaaccagagagggctcggctg$ |
| rs3823536 A>G | Forward gtgggtttgcaaggagacat  Reverse ctggagtcccaggagacagt  Probe TTACCTATGATTGATCGTGGTGATAggacTcactaggggaggaagtgc$ |
| rs3778753 G>A | Forward ttgattggggtggtctgaat  Reverse tccatacaggcagcttaggg  Probe TTACCTATGATTGATCGTGGTGATATCCGcaTgagctctaacccgaacagcatccaaactcc$ |
| rs11767834 C>T | Forward gtctcaagtgaagggccaag  Reverse ggaaacagaagccacagctc  Probe AATGAcagtgaagctAtggcctgggagg$ |
| rs13245639 C>T^a^ | Forward ttggctcattgcaaactctg  Reverse ttctcataggaggccaggtg  Forward gactacaggcacccaccatc  Reverse tcacacctgtaatcccagca  Probe TTACCcactttgggaggccaaggcggacagatc$ |
| rs4728141 C>T | Forward tgccaccacgatggtagata  Reverse ccaggagttcaaggttacgg  Probe TTACCTATGATTGATgatgaaccacagcactccagcctggaca$ |
| rs960633 G >T | Forward gggctcccgatgttacacta  Reverse tggagcgagtggataaggtt  Probe CCAGAccctggggggcctcaacattccttgctg$ |
| rs11982901 C>T | Forward caggtcattcaagggctgtt  Reverse taccatcgtggtggcaca  Probe TTACCTATGAatcacgccactgcactccagcctgggtg$ |
| rs1983607 C>T | Forward ggatgcatatttggttttagcttt  Reverse gctgtttacaggaggcacact  Probe TTACCctgagtagctgggattacagTtgtgtgccacca$ |
| rs729068 C>T | Forward caggacttgcactggggtat  Reverse cctcctgtctcttgcccata  Probe TTACCTATGATTGATCGTGGggtcagagaatgcaTacgcacaggtgtg$ |
| rs12706860 C>G | Forward ctcacctggagccacctagt  Reverse tccaagaagcatccttcacc  Probe ctgtctccaggggtttggtgatgtccag |
| rs754284 C>G | Forward ggggtgggtctatggatctc  Reverse gcctgacacacactgtggaa  Probe TTACCTATGATTGATaacccacatggctccctggctctgcctg$ |
| rs2402940 C>T | Forward tttgcttaggtacaaaggaaaatac  Reverse tttaagacgagcctggtcaa  Probe TTACCTATGATTGATCGTGGTGATAcctacttggaaggctgaggcaggagaatcactt |
| rs3778754 G>C | Forward gtgctgctctctgtccttcc  Reverse gcacctggctttgctgtatt  Probe TTACCTATGATTGATagtaggacctcaaatctcatttcctcgtctctaaaagg$ |
| rs7808659 C>A | Forward agggagggagagtggagaga  Reverse cctcatgatcctcctgcttc  Probe tgagccactgtacccggccctaagtaat |
| rs11763323 G >T | Forward taggagtttcggcaaaggtg  Reverse caggcccaagagaaatcaag  ProbeTTACCTATGATTGATCGTGGTGATATCCGagaaccgttcccatcacttcatgccgtccctta$ |
| rs3757385 G>T | Forward aggggaagtcaaggcagact  Reverse tctgaaccgttttcgattcc  Probe TTACCcaccttcacacacctcctgttccttctc$ |
| rs754281 C>T | Forward tgctccaggagagaaaggag  Reverse gggggcaggttcttactagg  Probe TTACCTATGAcctcgagacaggcatgggtcggtggaga$ |
| rs1874330 T>C | Forward atattccagccaggggaaat  Reverse aagggcaagtagaggggaag  Probe TTACCTATGATTGATCGTGGTGATAgaggcggtgggTacagggaggtctgtcc$ |

^a^ This SNP was amplified with a nested PCR

**Supplementary Table 3:** Primers that were used for genotyping the indicated polymorphisms in the healthy Spanish controls by Sanger sequencing.

| Polimorfismo | Oligonucleotides |
| --- | --- |
| rs3778752 T>G  and rs3778751 T>A | Forward tgattggggtggtctgaatta  Reverse cttttgcctgcagctaggtc |
| CGGGG indel | Forward cgccgtctggcatctccct  Reverse tgagctctgcccaggctgc |
