# Supplementary material for: Cis-regulation of IRF5 expression is unable to fully account for systemic lupus erythematosus association: analysis of multiple experiments with lymphoblastoid cell lines
Source: Arthritis Res Ther. 2011 May 31;13(3):R80. doi: 10.1186/ar3343 (PMC3218890; doi:10.1186/ar3343)
Supplement: Additional file 2 — Supplementary results. Complementary analyses of the IRF5 lineal regression models and of the haplotype distribution, together with linkage disequilibrium maps and expression results pertaining to probes targeting less representative IRF5 exons. [file ar3343-S2.DOC]

**Supplementary Table 1:** Parameters of the multiple linear regression models including known functional polymorphisms to account for IRF5 expression in each of the experimental data sets. Models including exon 6 indel are not shown because there is not any suggestion it could play a role in IRF5 expression. Parameters indicating the fit of the model and the *P* values corresponding to the independent contribution of each polymorphism to models are shown. Expression data sets are labeled as in Table 2.

| Data set | Polymorphisms in model | Adjusted r^2^ | Model *P* | Polymorphism’s *P*-value in model |
| --- | --- | --- | --- | --- |
| K8 | rs10954213 | 0.21 | 2.3 x10^-4^ |  |
|  | rs2004640 | 0.14 | 0.0023 |  |
|  | CGGGG indel | 0.13 | 0.0034 |  |
|  | rs10954213  rs2004640 | 0.2 | 9.7 x 10^-4^ | 0.031  n.s. |
|  | rs10954213  CGGGG indel | 0.19 | 0.0012 | 0.027  n.s. |
|  | rs2004640  CGGGG indel | 0.13 | 0.0098 | n.s.  n.s. |
|  |  |  |  |  |
| S | rs10954213 | 0.75 | 3.3 x10^-19^ |  |
|  | rs2004640 | 0.47 | 7.2 x10^-10^ |  |
|  | CGGGG indel | 0.58 | 9.5 x10^-13^ |  |
|  | rs10954213  rs2004640 | 0.76 | 1.5 x10^-18^ | 1.4 x10^-11^  n.s. |
|  | rs10954213  CGGGG indel | 0.76 | 2.2 x10^-18^ | 1.3 x10^-8^  n.s. |
|  | rs2004640  CGGGG indel | 0.57 | 2.4 x10^-11^ | n.s.  3.5 x10^-4^ |
|  |  |  |  |  |
| C | rs10954213 | 0.52 | 1.3 x10^-10^ |  |
|  | rs2004640 | 0.33 | 1.4 x10^-6^ |  |
|  | CGGGG indel | 0.36 | 3.7 x10^-7^ |  |
|  | rs10954213  rs2004640 | 0.52 | 7.7 x10^-10^ | 1.5 x10^-5^  n.s. |
|  | rs10954213  CGGGG indel | 0.51 | 1.3 x10^-9^ | 9.6 x10^-5^  n.s. |
|  | rs2004640  CGGGG indel | 0.35 | 2.4 x10^-6^ | n.s.  n.s. |
|  |  |  |  |  |
| D | rs10954213 | 0.2 | 1.3 x10^-10^ |  |
|  | rs2004640 | 0.21 | 4.9 x10^-11^ |  |
|  | CGGGG indel | 0.23 | 3.7 x10^-12^ |  |
|  | rs10954213  rs2004640 | 0.27 | 1.6 x10^-13^ | 7.2 x10^-5^  2.6 x10^-5^ |
|  | rs10954213  CGGGG indel | 0.27 | 4.0 x10^-13^ | 0.0028  6.7 x10^-5^ |
|  | rs2004640  CGGGG indel | 0.23 | 1.8 x10^-11^ | n.s.  0.011 |

**Supplementary Table 2:** Description of the IRF5 haplotypes as defined with tagSNPs in Ferreiro-Neira et al. [4].

|  |  | | | | | | | | | |
| --- | --- | --- | --- | --- | --- | --- | --- | --- | --- | --- |
| Haplotype | rs729302 | rs2004640 | rs752637 | Exon 6 indel | rs10954213 | rs13242262 | rs10488630 | rs10488631 | rs2280714 | rs4731535 |
| 1 | C | G | G | de | A | T | A | T | A | C |
| 2 | C | G | A | in | G | A | A | T | G | T |
| 3 | A | G | A | in | G | A | A | T | G | T |
| 4 | A | T | G | de | A | T | G | T | A | C |
| 5 | A | T | G | de | A | T | A | T | A | C |
| 6 | A | T | G | in | A | T | A | C | A | T |

**Supplementary Table 3:** Lack of correlation between SLE-associated haplotypes defined as in Ferreiro-Neira et al. [4] and the best linear regression model for each of the IRF5 expression data sets. Spearman’s rank order r_s_^2^ values are given for the carriage of protective haplotypes H1 and H2 and for the risk haplotype H6 taken individually and for the diplotypes made of protector, neutral and risk haplotypes.

| Data set | H1 | H2 | H6 | Diplotypes |
| --- | --- | --- | --- | --- |
| K8 | 0.0001 | 0.04 | 0.02 | 0.0005 |
| S | 0.08 | 0.03 | 0.04 | 0.003 |
| C | 0.008 | 0.03 | 0.03 | 0.12 |
| D | 0.15 | 0.06 | 0.02 | 0.06 |

**Supplementary Figure 1:** Maps of LD in the *IRF5* block representing pairwise A) D’ values and B) r^2^ values. Positions of the *IRF5* gene and of rs729302 are highlighted.

**A.**

**
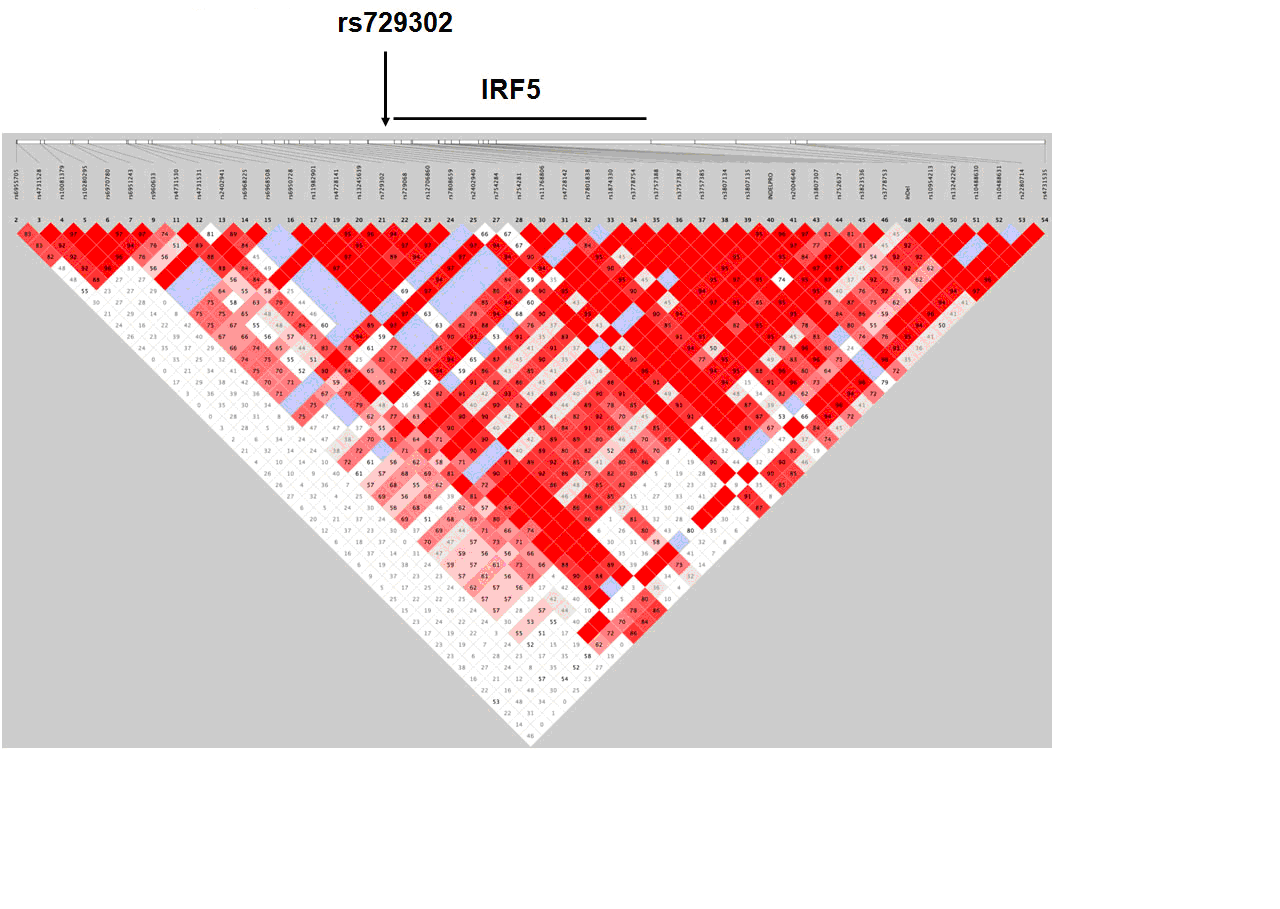
**

**B.**

**
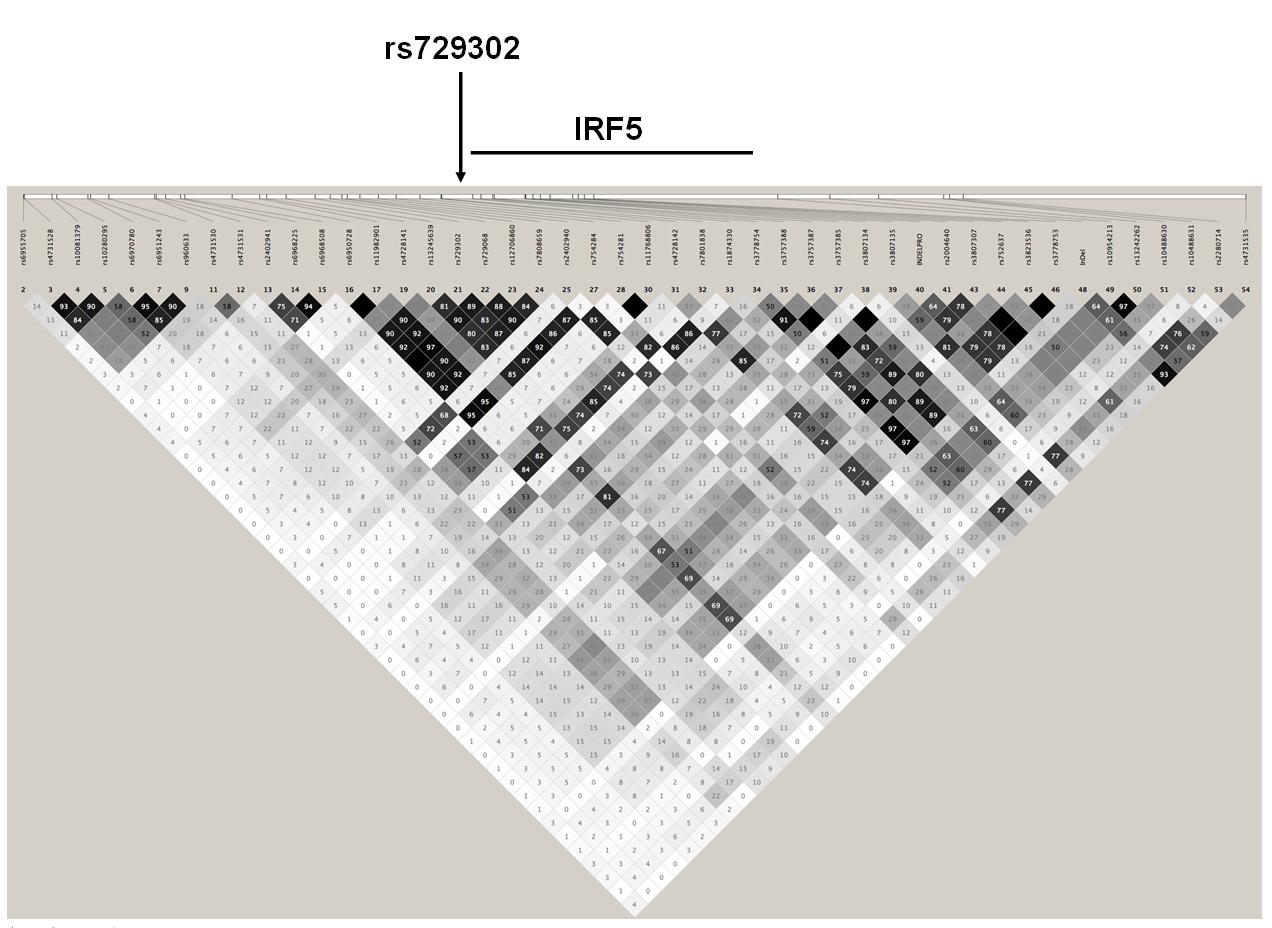
**

**Supplementary Figure 2:** Pairwise r^2^ between the most relevant polymorphisms in our analysis (detail from Supplementary Figure 1B).


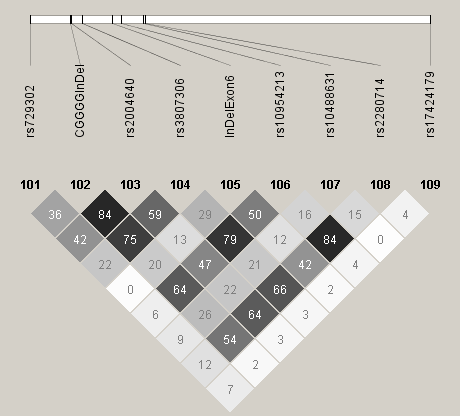


**Supplementary Information:**

**Expression of the IRF5 isoforms:** Some of the studies in our analysis had included probes targeting IRF5 isoforms (Figure 1 in the main text). Expression results from the probe targeting exon 1C did not show association with any of the IRF5 polymorphisms, suggesting that its alternative splicing is not cis-regulated. This was in contrast with a recent report showing association of rs10488631 with expression of this exon in SLE blood cells [26]. Two of the studies contained a probe targeting the 3’UTR posterior to rs10954213. Presence of this part of the 3’UTR was largely dependent on this SNP (with *P* values 9.7 x10^-21^ in Kwan’s data and 1.4 x10^-33^ in Dixon’s data) as has already been described [3,23]. No meaningful analysis of expression data for exon 1A was possible given the widely discordant results obtained in the two studies targeting this exon (Figure 2 in the main text). Also, no analysis was done for the probe in variant 5 of exon 6 given its very low expression level.
